# Single-tube multiplex PCR using type-specific *E6/E7 *primers and capillary electrophoresis genotypes 21 human papillomaviruses in neoplasia

**DOI:** 10.1186/1750-9378-6-1

**Published:** 2011-01-17

**Authors:** Michael Dictor, Janina Warenholt

**Affiliations:** 1 University and Regional Laboratories Region Skåne, Department of Pathology, Lund University Hospital, Sölvegatan 25, SE 22185, Lund, Sweden

## Abstract

**Background:**

Human papillomavirus (HPV) *E6/E7 *type-specific oncogenes are required for cervical carcinogenesis. Current PCR protocols for genotyping high-risk HPV in cervical screening are not standardized and usually use consensus primers targeting HPV capsid genes, which are often deleted in neoplasia. PCR fragments are detected using specialized equipment and extra steps, including probe hybridization or primer extension. In published papers, analytical sensitivity is typically compared with a different protocol on the same sample set.

A single-tube multiplex PCR containing type-specific primers was developed to target the *E6/E7 *genes of two low-risk and 19 high-risk genotypes (HPV6, 11 and 16, 18, 26, 31, 33, 35, 39, 45, 51, 52, 53, 56, 58, 59, 66, 68, 70, 73 and 82) and the resulting short fragments were directly genotyped by high-resolution fluorescence capillary electrophoresis.

**Results:**

The method was validated using long oligonucleotide templates, plasmid clones and 207 clinical samples of DNA from liquid-based cytology, fresh and formalin-fixed specimens and FTA Microcards^® ^imprinted with cut tumor surfaces, swabbed cervical cancers or ejected aspirates from nodal metastases of head and neck carcinomas. Between one and five long oligonucleotide targets per sample were detected without false calls. Each of the 21 genotypes was detected in the clinical sample set with up to five types simultaneously detected in individual specimens. All 101 significant cervical neoplasias (CIN 2 and above), except one adenocarcinoma, contained *E6/E7 *genes. The resulting genotype distribution accorded with the national pattern with HPV16 and 18 accounting for 69% of tumors. Rare HPV types 70 and 73 were present as the sole genotype in one carcinoma each. One cervical SCC contained DNA from HPV6 and 11 only. Six of twelve oropharyngeal cancer metastases and three neck metastases of unknown origin bore *E6/E7 *DNA; all but one were HPV16. One neck aspirate contained atypical squames with HPV26.

Analytical sensitivity in dilute plasmid mixes was variable.

**Conclusions:**

A primer-rich PCR readily detects the *E6/E7 *oncogenes of 21 HPV types in cellular and fixed tissue specimens. The method is straightforward, robust and reproducible and avoids post-PCR enzymatic and hybridization steps while detecting HPV with high clinical sensitivity in significant HPV-related neoplasia regardless of specimen type.

## Background

Alpha-human papillomaviruses (HPV) are sexually transmitted and may infect the anogenital and oral mucosae. They either resolve without sequelae or cause condyloma (HPV types 6 and 11 and their relatives) or rarely carcinoma, particularly cervical cancer and its precursors (cervical intraepithelial neoplasia, CIN). HPV16 and 18 account for 70% to 80% of cervical cancers with the remainder due to other alpha- HPV genotypes. HPV are labeled high-risk (HR-HPV) if found in cancers in disproportion to their prevalence; uncertainty exists as to whether certain genotypes (e.g., HPV26, 53, 70, 73 and 82) should be classified as high-risk.

Cervical carcinogenesis depends on the deregulation of HPV *E6/E7 *in cell nuclei as dysplasia progresses. Although no "gold standard" for type-specific HPV genotyping has been agreed upon, current methods generally employ the polymerase chain reaction (PCR) with consensus primers aimed at the *L1 *capsid gene [1], despite the fact that it is the *E6/E7 *genes which are most reliably conserved in cervical cancer [2]. The type-specific *E6/E7 *junction is also less prone to sequence variation than other genes, which like the capsid genes may be lost during integration of viral DNA into the host cell genome [2-5].

By combining labeled *E6/E7 *primers in a multiplex PCR (MPCR) to yield products which are size and color-specific for each HPV genotype on capillary electrophoresis, the need for nested PCR, amplicon sequencing, probe hybridization or enzymatic detection steps [6] and specialized equipment is obviated. MPCR targeting these type-specific oncogenes has been reported but the methods may use proprietary technology not widely available and require additional post-PCR treatments, making them less suitable for small specimen volumes in the pathology laboratory [6,7]. Capillary electrophoresis, on the other hand, is well established, widely available for fragment size analysis and it yields reproducible size peaks with a tolerance of ± 0.5 nucleotide (nt). We describe a single-tube straightforward *E6/E7 *MPCR capable of detecting 19 HR-HPV in a test set of highly significant lesions (CIN 2 to carcinoma) submitted as cytology specimens, including aspirates from metastases, as well as fresh and formalin-fixed tumor embedded in paraffin (FFPE).

## Results

We targeted in a single microfuge tube 19 definite or probable HR-HPV genotypes (HPV types 16, 18, 26, 31, 33, 35, 39, 45, 51, 52, 53, 56, 58, 59, 66, 68, 70, 73, 82) [8,9] and in addition low-risk (LR) HPV6 and 11 and as a control the human β-globin gene. Table 1 lists the accession numbers of the HPV targets in GenBank, primer sequences, labels, true amplicon sizes and their observed electrophoretic sizes. Template DNA came from five sources: (i) long oligonucleotides mimicking *E6/E7 *targets (except for HPV types 16, 51 and 73) for determination of electrophoretic product size, (ii) for analytical sensitivity a test panel of 14 plasmid-cloned HPV genotypes [10], courtesy of the WHO HPV Reference Lab, Malmö, Sweden, (iii) for clinical sensitivity, DNA extracted from 500 μL of pelleted cellular material in PreservCyt solution^® ^used for ThinPrep cytology (Cytyc, USA), which specimens were obtained with a cytobrush (N = 97, including 37 consecutive normal, 13 atypical, 39 dysplasias and 9 cancers), (iv) FFPE specimens (N = 44, including 11 cervical dysplasias and 33 cancers) and (v) Indicating FTA Microcards^® ^(Whatman, Germany) with imprinted cut tumor surface or applied swabs/aspirates (N = 64, including 30 primary cervical carcinomas and 3 nodal metastases, in addition to 31 aspirates of nodal metastases from head and neck SCC). FTA cards significantly expedite sample collection, storage and preparation and have been described previously in lymphoma MPCR [11]. Patient specimens were de-identified to conform to the ethical guidelines established at our institution.

**Table 1:** HPV primer and product characteristics

HPV type	**Genbank accession no**.		E6/E7 Primers^1^		Product's molecular size (nt)	Product's electrophoretic size (nt)^2^
		**Forward**	**5' label**	**Reverse**		
39	M62849	ACA GTG TCG ACG GTG CTG GA	FAM	GCT TTG GTC CAC GCA TAT CTG A	94	92
70	U21941	ACA GTG CCG ACA CTG CTG GA	NED	GGC CGT GGT CCA TGC ATA TT	95	94
68	Y14519	ACA GTG TCG **S**CA CTG CTG GA	HEX	GGG CTT TGG TCC ATG CAT AGT	95	95
18	X05015	AGT GCC ATT CGT GCT GCA AC	FAM	ATG TTG CCT TAG GTC CAT GCA T	98	96
56	X74483	TGG TTG GAC CGG GTC ATG T	HEX	CGT CTT GCA GCG TTG GTA CTT T	103	100
45	X74479	GGA CAG TAC CGA GGG CAG TGT A	NED	CCG GGG TCC ATG CAT ACT TAT	107	106
33	M12732	AAT ATT TCG GGT CGT TGG GC	FAM	AAC GTT GGC TTG TGT CCT CTC A	109	107
35	M74117	GGT GGA CAG GTC GGT GTA TGT C	HEX	GTT GCC TCG GGT TCC AAA TC	120	116
66	U31794	ACC GGG TCA TGT TTG CAG TGT	NED	CGT TTG CGG TGC AAG TTC TAA T	122	120
6/6b	AF092932 X00203	CCA AGG C**R**C GGT TCA TAA AGC	FAM	GGG TCT GGA GGT TGC AGG TCT A	123	120
6a	L41216	CCA AGG CGC GGT TTA TAA AGC	FAM	GGG TCT GGA GGT TGC AGG TCT A	123	120
31	J04353	GTG GAC AGG ACG TTG CAT AGY A	HEX	GGT CAG TTG CCT CAG GTT GCA	124	121/203^3^
26	X74472	GGG CAG TGG AAA GGG TTG TGT	NED	GGT TGC GGC ACC AGA TCT AGT A	128	126
51	M62877	AAT GCG CTA ATT GCT GGC AA	HEX	TGC TCG TAG CAT TGC AAG TCA A	143	142
53	X74482	ATA TGT GGA CCG GGT CGT GC	FAM	GGC ATT GCA GGT CAA TCT CAG T	143	143
82	AB027021	ACG GGA CAG TGT GCA AAT TGC	HEX	TGC TCG TAG CAT TGC AAG TCA A	150	150
52	X74481	GTT GGA CAG GGC GCT GTT C	NED	CCT CCT CAT CTG AGC TGT CAC C	166	167
59	X77858	ACA GTG TCG TGG GTG TCG GA	FAM	TGC TCG TAG CAC ACA AGG TCA A	169	169
58	D90400	AGG GCG CTG TGC AGT GTG T	HEX	CAT CCT CGT CTG AGC TGT CAC A	172	172
11	M14119	GGG AAA GGC ACG CTT CAT AAA	NED	TGT CCA CCT TGT CCA CCT CAT C	190	190
16	K02718	GTG GAC CGG TCG ATG TAT GTC T	HEX	TCC GGT TCT GCT TGT CCA GC	209	210
73	X94165	AAC AGT GGA CCG GAC GCT GT	FAM	GGC AAG GCA TAC TGT GCA CTG A	258	260
β-globin	NG000007	GAA GAG CCA AGG ACA GG TAC	HEX	CAA CTT CAT CCA CGT TCA CC	268	268

^1 ^Each ambiguous base code S, R and Y yields two different primer sequences. HPV types 51 and 82 share identical reverse primers.

^2 ^Default colors: FAM, blue; NED, black; HEX, green.

^3 ^Secondary HEX peak at 203 nt is due to cross-annealing to the HPV16 reverse primer.

**Figure 1:**
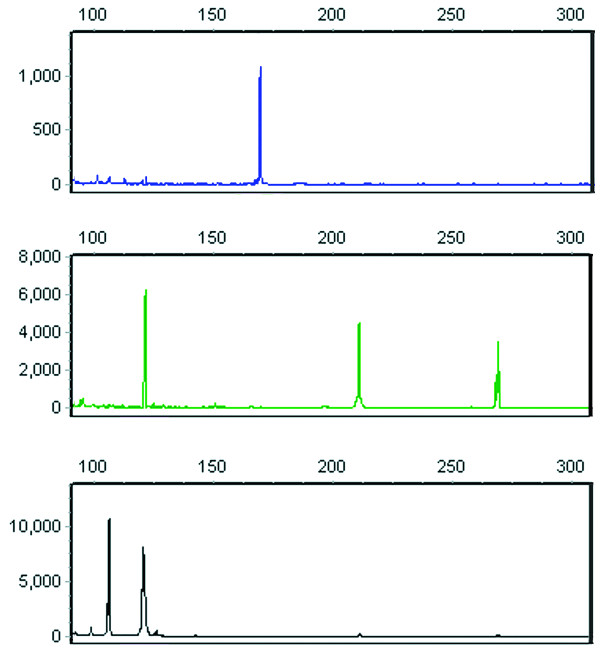
**MPCR electropherogram in multiple infection**. An autoscaled electropherogram of a single CIN 3 sample contained HPV *E6/E7 *types 16, 31, 45, 59 and 66, which are resolved in three panels, each representing a separate color channel (a red size marker channel is not shown). The X-axis indicates product size in nucleotides and the Y-axis shows fluorescence intensity in arbitrary units. The upper panel contains a single blue peak at 169 nt, corresponding to HPV59. In the middle panel, green peaks at 121, 210 and 268 nt confirm HPV31, HPV16 and the β-globin control, respectively. The bottom panel's black peak at 106 nt indicates HPV45 and the second peak at 121 nt matches HPV66. In the routine electropherogram with composite channels, peaks for types 31 and 66 overlap but are easily identified by color.

Results in clinical material are presented in Table 2. All 19 HR-HPV were detected in at least one and most in more than two clinical samples with as many as five genotypes observed in a single specimen (Figure 1). Peak amplitudes almost always exceeded 1000 fluorescence units. LR-HPV type 6 was present in five samples, one of which, a cervical squamous cell carcinoma (SCC), uniquely contained the *E6/E7 *genes from types 6 and 11 but not from any HR-HPV. This was the only specimen to contain HPV11.

**Table 2:** HPV types in clinical samples

					LR-HPV		HR-HPV																		
	*N*	β-globin	HR-HPV (%)	> 1 genotype (%)	6	11	16	18	26	31	33	35	39	45	51	52	53	56	58	59	66	68	70	73	82
																									
**Cervical cytology**																									
normal	37	37	9 (24%)	3 (8%)	1	0	1	2	0	0	0	0	1	0	0	1	0	3	1	0	1	2	1	0	0
ASCUS^1^	12	12	6 (50%)	3 (25%)	0	0	0	3	0	0	1	0	1	3	1	1	0	0	0	0	0	0	0	0	1
columnar atypia	1	1	1 (100%)	0	0	0	0	1	0	0	0	0	0	0	0	0	0	0	0	0	0	0	0	0	0
CIN 1	24	24	18 (78%)	6 (25%)	2	0	4	2	0	2	3	1	1	2	2	1	0	1	1	2	1	1	1	2	0
CIN 2	11	11	11 (100%)	4 (36%)	0	0	7	3	0	2	2	0	0	0	0	0	0	0	1	0	1	0	0	0	1
CIN 3	4	4	4 (100%)	2 (50%)	0	0	3	0	0	1	0	0	0	1	0	0	1	0	1	1	1	0	0	1	0
SCC^2^	9	9	9 (100%)	3 (33%)	0	0	3	1	0	2	2	0	0	1	0	2	0	1	1	1	1	0	0	0	0
*subtotal*	*98*	*98*	*58*	*21*	*3*	*0*	*18*	*12*	*0*	*7*	*8*	*1*	*3*	*7*	*3*	*5*	*1*	*5*	*5*	*4*	*5*	*3*	*2*	*3*	*2*
																									
**FFPE**																									
CIN *NOS*	11	5	11 (100%)	2 (18%)	1	0	3	2	0	1	0	0	1	0	1	1	1	0	0	0	0	0	0	1	0
SCC, cervix	23	13	23 (100%)	2 (9%)	0	0	14	2	0	2	0	0	0	1	0	0	0	2	0	1	0	0	0	0	0
ADCA, cervix	10	5	9 (90%)	1 (10%)	0	0	5	3	0	0	0	0	0	1	0	0	0	0	0	1	1	0	0	0	0
																									
*subtotal*	*44*	*23*	*43*	*5*	*1*	*0*	*22*	*7*	*0*	*3*	*0*	*0*	*1*	*2*	*1*	*1*	*1*	*2*	*0*	*2*	*1*	*0*	*0*	*1*	*0*
																									
**FTA**																									
SCC^3^	23	23	22 (96%)	1 (4%)^4^	1	1	14	1	0	1	1	1	0	2	0	1	1	1	1	0	0	0	1	1	0
ADCA^5^	11	11	11 (100%)	1 (9%)	0	0	5	4	0	2	0	0	0	0	0	0	0	0	0	1	0	0	0	0	0
SCC neck metastasis	31^6^	31	10 (100%)^7^	0	0	0	9	0	1	0	0	0	0	0	0	0	0	0	0	0	0	0	0	0	0
																									
*subtotal*	*65*	*65*	*44*	*2*	*1*	*1*	*28*	*5*	*1*	*3*	*1*	*1*	*0*	*2*	*0*	*1*	*1*	*1*	*1*	*1*	*0*	*0*	*1*	*0*	*0*

**total**	**207**	**186**	**145**	**29**	**5**	**1**	**68**	**24**	**1**	**13**	**9**	**2**	**4**	**11**	**4**	**7**	**3**	**8**	**6**	**7**	**6**	**3**	**3**	**5**	**2**

^1 ^Atypical squamous cells of undetermined significance.

^2 ^One bronchoalveolar lavage for a HPV59^+ ^SCC metastasis from cervix cancer is included.

^3 ^Includes 5 FTA imprints from consecutively resected cervical SCC (3 primary, 2 metastatic nodes) and 18 consecutive surface tumors swabbed *in vivo*.

^4 ^Both low-risk HPV types 6 and 11 E6/E7 were present in a cervical SCC from an elderly woman.

^5 ^Includes 6 FTA imprints from consecutively excised tumors (5 primary, 1 metastatic node) and 5 consecutive ADCA swabbed *in vivo*.

^6 ^Primary tumors were located in the oropharynx (11), mesopharynx (2), nasopharynx (3), tongue (1), larynx (3), oral cavity (1), paranasal sinus (1), preauricular skin (3) and unknown sites (6).

^7 ^HPV^+ ^metastases were derived from 6 primary tumors in the oropharynx (5 tonsillar and 1 base of tongue) and 4 unknown sites. HPV26 was found in atypical squames in a neck node without a confirmed diagnosis of carcinoma but is included among SCC of unknown origin.

About one-fourth of 37 consecutive samples with normal cytology were infected with HR-HPV and three of these (8%) had multiple genotypes. In 15 consecutive exfoliative samples of high-grade CIN (grades 2 and 3), one or more HR-HPVs occurred in all in the following order of frequency (slash indicates equal frequencies): types 16, 18/31, 33/58/66 and 45/53/59/73/82.

HPV *E6/E7 *genes were detected in 75 of 76 primary or metastatic cervical carcinomas (21 adenocarcinomas [ADCA], 55 SCC, including the single SCC containing LR-HPV only), as single infections in 91%. The one negative sample was a β-globin^+ ^ADCA (de-identification prevented confirmation of a true primary cervical tumor in this case). HPV16 and 18 accounted for 69% of the 75 HPV-associated cervical cancers, and other genotypes were in order of frequency: 31, 45, 56/59, 33/52, 58/66 and 35/53/70, which accords with the national statistics [12].

Coinfection with more than one HPV type occurred less often in carcinomas in contrast to dysplastic cytology specimens, consistent with decreased rates of multiple infections among the generally older patients with carcinoma.

Nine of 31 nodal aspirates of SCC metastases from head and neck tumors (Table 2 footnotes 6 and 7) contained HPV. The primary site in HPV^+ ^tumors was oropharyngeal in six instances, consistent with previous studies [13], and the origin was unknown in three. All nine aspirates produced prominent HPV16 peaks only, confirming the overwhelming preponderance of this genotype and the reported lack of HPV18 in oropharyngeal SCC [14]. HPV26, a suspected HR-HPV [8] previously reported in extracervical SCC [15], was unexpectedly detected as the sole type in one nodal aspirate containing atypical squames but without a confirmed cancer diagnosis. This result was reconfirmed in a singleplex PCR on the same aspirate using HPV26 primers.

HR-HPV26, 39, 51, 68 and 82 were not present in this series of cervical carcinomas. Types 26, 53, 70, 73 and 82 are often not included in PCR protocols, partly due to inconclusive evidence of an overincidence in cancer. In our series, these types occurred 14 times *in toto*. Among cervical SCC, HPV types 70 and 73 each occurred once, type 53 was found in one SCC together with type 45, while type 26 was not detected in any cervical material. The rationale for inclusion of type 70 in the MPCR as a HR-HPV is further bolstered by its genotypic and biologic relation with HR-HPV [9,16]. Among cell samples with high-grade CIN, one harbored HPV82, 31 and 33 concomitantly and another contained HPV53, 58 and 73.

In contrast to MPCR's high clinical sensitivity in lesional samples, its analytical sensitivity in dilute plasmid-HPV templates was variable, partly depending on whether single or multiple targets were present. Generally, six of the eight most prevalent HPV types needed >100 genome-equivalents/μL to elicit a definitive peak, especially if mixed with other plasmids, while half of the remaining genotypes were detected at a concentration 10-fold less (data not shown).

The MPCR was robust in clinical samples, producing readily interpretable peaks and results were reproducible on repeat runs. An occasional small spurious peak due to saturation of the laser detector disappeared after diluting the product.

Secondary peaks attributable to primer cross-annealing appeared occasionally, mainly in cervical cytology specimens (presumably the result of higher virus loads in productive infections), but did not correspond in size and color to true peaks and had amplitudes usually below our cutoff. In theory, mispriming of a forward primer could result in an additional peak for a given HPV type, while a mispriming reverse primer could present a new peak of the same or different size and color (all fluorophores were regularly placed on the reverse primer) as the target peak.

Forward primers in types 39, 59, 68 and 70, which are all 20-mers and share 3 to 7 nt in their 3' end sequence, might offer potential mispriming among their intended targets, which would not necessarily produce a peak shift related to anomalous product sequence and none was seen in electropherograms with these genotypes. Moreover, use of the "touchdown" technique in initial PCR cycles probably abrogated any tendency of this limited sequence similarity to competitively inhibit visualization of mixed infection. For example, two mixed HPV68/70 infections were easily detected.

*In silico *analysis of individual E6/E7 target sequences queried for significant complementarity (> 85% overall complementarity with a requirement for complete complementarity in the last three 3' bases) with any of the primers in the master mix yielded 29 potential misprimed products of varying size and color derived from 16 of the 21 genotypes when testing clinical material, none of which were of the same size but different color from that expected for the target. Complete complementarity was noted only for HPV31, where its forward primer amplified type 31 E6/E7 in conjunction with the predicted homologous type 16 reverse primer. Thus, HPV31 gave the most significant and consistent secondary peak (Table 2 footnote 4). Other predicted spurious amplicons included HPV66 annealing with its legitimate forward primer and type 56 reverse primer (91% complementarity) to yield a green product 1 to 2 nt larger than that of HPV68. Nonetheless, in no case was an apparent type 68 peak (three instances) found together with type 66 (six instances). HPV35, if annealed to the 52 reverse primer (91% complementarity) could yield an amplicon similar to HPV52 but this combination of products was not seen either.

## Discussion

We developed a primer-rich MPCR which offers single-tube economy and straightforward simplicity in genotyping 21 potentially oncogenic HPV using type-specific *E6/E7 *primers. This broad spectrum and the short resulting amplicons necessary for formalin-degraded tumor specimens yielded a clinical sensitivity of 100% for high-grade CIN and 99% for cervical carcinoma, which clearly exceeds the HR-HPV detection rate reported for general primers when product detection is limited to the most common genotypes [17]. The reasons for failure of consensus capsid primers to amplify their target include relatively frequent base substitutions and deletions which may affect primer annealing or probe hybridization [2,18-20]. Importantly, our MPCR targets the *sine qua non *of cervical tumorigenesis, the *E6/E7 *oncogenes [21,22]. Selection pressure appears to be minimal in codons within the *E6/E7 *transition zone and sequence variations in the HPV16 *E6 *gene, for example, tend largely to involve single nucleotides upstream of the primer region we used in all 21 genotypes [21].

Cervical cancer cell nuclei contain viral DNA either integrated in the host cell genome, as free episomes or both. If viral episomes disappear, which occurs in 20% to 50% or more of tumor specimens, most consistently in HPV18-induced tumors [18], only residual integrated HPV DNA would remain in the host cell and this would include the obligate *E6/E7 *oncogenes [2,4]. A recently reported multiplex PCR targeting the *E7 *oncogene showed greater analytical sensitivity than PCR with broad-spectrum consensus *L1 *primers where product detection in both analyses used bead-based hybridization (Luminex) [23]. In the single SCC in which our MPCR detected both HPV6 and 11 (footnote 2, Table 2), general *L1 *primers (GP5+/6+) in a PCR on the same patient sample followed by bead-based hybridization typing identified HPV6 but failed to identify HPV11, whereas a singleplex PCR with our type 11 *E6/E7 *primers repeatedly confirmed the original result.

When compared with cytology, HPV genotyping by PCR offers higher sensitivity but lower specificity in detecting intraepithelial neoplasia [24] and PCR genotyping has been introduced as a means of triaging in cervical cancer screening programs. Ultimately, molecular methods may replace cytologic screening, in which case longitudinal testing using different PCR protocols may be expected to yield conflicting results. This makes a single, uniform, highly sensitive and reliable platform desirable for use in all relevant specimens, regardless of where in the diagnostic chain the specimen is obtained: screening, tissue biopsy/resection and eventual confirmation of metastasis by aspiration cytology or excision [25-28].

Our detection of rare HR-HPV in high-grade CIN and several suspected HR-HPV types in carcinoma points out that a relatively broad-spectrum multiplex PCR capable of typing all cervical carcinomas offers a desirable level of coverage but could still miss the rare tumor related to a low-risk HPV not covered in the analysis. In this regard, primers for additional genotypes could probably be added to the 46 already present in our MPCR without adversely affecting efficiency.

Although multiplex PCR with capillary electrophoresis gives lower analytical sensitivity, which would militate against its use in detecting infection per se or minimal residual malignant disease (cf., lymphproliferative diseases), in detection scenarios in the present study clinical sensitivity is more relevant and this was retained. If appropriately automated, scaled and tested on a larger population sample, our MPCR might be less likely to overburden triaging schemes with excessive detection of infections destined to resolve [29].

The cost of materials for the MPCR (unoptimized for use in screening) including primers and reagents, was about USD 7 per sample as of the year 2008.

## Conclusions

A primer-rich PCR readily detects the *E6/E7 *oncogenes of 21 HPV types in fresh and fixed cytology and tissue specimens. The method is straightforward, robust, avoids post-PCR sequencing and hybridization steps by using fragment size analysis and detects HPV with high sensitivity in significant HPV-related neoplasia regardless of specimen type.

## Methods

### Primer and product details

Primer parameters were adjusted to yield as many amplicons separated in size by ~3 or more nucleotides as possible while maintaining high PCR efficiency. Primer pairs were chosen to avoid potential duplex formation within and between pairs. Each primer pair with amplicon was used in a BLAST query of human and viral databases http://www.ncbi.nlm.nih.gov/tools/primer-blast/ to confirm the sequence's uniqueness for each HPV genotype and to adjust primers for minor polymorphisms in primer binding sites reported for subtype variants. All reverse primers were synthesized with 5' labels (FAM, blue; HEX, green or NED, black), which were alternated with each increase in corresponding amplicon size. Primers for the human β-globin control gene were designed to yield a green 268 nt product. The result was 46 unique primers designed to yield short products suitable for both high-molecular weight DNA and formalin-fixed material; expected HPV product sizes ranged from 94 to 258 nt with all but two being≤190 nt. Primer sets were then synthesized and purified by HPLC (Eurofins MWG/Operon, Germany).

### PCR protocol

DNA was extracted from sections and from cytology fluid using a kit (GentraSystems, USA) according to the manufacturer's instructions. DNA was precipitated in isopropanol, the pellet resuspended in 10 mM Tris-buffer, pH 8.5 and 50-100 ng was used as template.

After extensive testing in >400 PCR using single and combined primer pairs, an optimal MPCR, capable of detecting at least 5 genotypes simultaneously in a single tube, was determined. It used 25 μL of (obligatory) Multiplex PCR Kit^® ^(cat. no. 206143, Qiagen, Germany) in a 50 μL reaction, which included 5 μL of a solution containing 0.3 μM of each forward and reverse primer for β-globin and HPV types 16, 39, 66 and 68 with 0.2 μM for all other primers. After 15 min at 95°C, 8 "touchdown" cycles (1°C decline in annealing temperature for each succeeding cycle) were run starting with annealing at 65°C for 90 s in the first cycle, then 95°C, 30 s; 65°C -1°C, 90 s for seven cycles; 72°C, 45 s, and 22 cycles with constant parameters (95°C, 30 s; 57.5°C, 90 s; 72°C, 45 s) with a final extension at 68°C for 15 min. All *E6/E7 *oligonucleotide targets were detected singly and in mixed combinations of 4 or 5 genotypes at estimated concentrations of 1.5 × 10^3 ^to 1.5 × 10^5 ^virus equivalents per genotype per μL.

### Capillary electrophoresis

One μl of PCR product was added to a mixture of 12 μl formamide and 0.5 μl ROX 500 size standard prior to heat-denaturation and loading on an ABI Prism^® ^3130 Genetics Analyzer whose capillaries were filled with POP7 polymer (minimum runtime 18 min). Data files were analyzed in GeneMarker^®, ^vers. 1.75. Peaks were called if they were ~1 nt or less in width, corresponded to the predetermined size (± 0.5 nt) and color of a given genotype and were ≥500 fluorescence units to allow ready distinction from baseline.

## Competing interests

The authors declare that they have no competing interests.

## Authors' contributions

MD designed the primers, the approach to validation, including the selection of appropriate materials and drafted the manuscript. JW designed the MPCR protocol and performed the analyses. Both authors collaborated in the interpretation of data for each sample. Both authors read and approved the final manuscript.
